# Vision and Cognitive Function in the Mid- to Later-Life Transition: the Study of Women’s Health Across the Nation

**DOI:** 10.1093/geroni/igaa057.3300

**Published:** 2020-12-16

**Authors:** Ajay Kolli, Michelle Hood, Carrie Karvonen-Gutierrez, Sayoko Moroi, Joshua Ehrlich, Brenda Gillespie, Sarah Wood, David Musch

**Affiliations:** 1 University of Michigan, Ann Arbor, Michigan, United States; 2 University of Michigan School of Public Health, Ann Arbor, Michigan, United States; 3 The Ohio State University College of Medicine, Columbus, Ohio, United States

## Abstract

In this analysis of data from the Study of Women Across the Nation (SWAN), a longitudinal cohort study of mid-life women, we sought to describe the relationship between vision impairment (VI) during mid-life and future CF during the mid- to later-life transition period. Presenting visual acuity was assessed at baseline, between 1996-2000, using a Titmus occupational screener. “Mild” and “moderate to severe” VI were respectively categorized as visual acuity <20/40 and <20/60 in the better seeing eye. CF was measured at near-annual visits from 2003-2015 using the East Boston Memory Test immediate (EBMTi) and delayed (EBMTd) recall and Digit Span Backwards (DSB). Linear mixed models with a random intercept and slope for age were constructed to detect associations between vision and CF, adjusting for race, education, financial strain, alcohol use, and tobacco use. The sample included 341 women aged 42-53 years (mean: 46.6) at baseline with up to 16 years of follow-up. The prevalence of VA <20/40 and <20/60 was 12.9% and 7.6%, respectively. While mild VI was associated with lower EBMTi (β= -0.46, p=0.034), EBMTd (β= -0.49, p=0.031), and DSB (β= -0.78, p= 0.015) scores in unadjusted models, these relationships were not statistically significant after adjustment for covariates. After adjusting for covariates, baseline moderate to severe VI was associated with worse EBMTi (β= -0.87, p=0.001) and EBMTd (β= -0.52 p=0.039) scores. Altogether, moderate to severe VI during mid-life was associated with lower verbal episodic memory scores (EMBTi/d) over a 16-year period representing the transition from mid-life to later adulthood.

